# A single-center study of 100 consecutive patients with localized prostate cancer treated with stereotactic body radiotherapy

**DOI:** 10.1186/1471-2490-13-49

**Published:** 2013-10-17

**Authors:** Giampaolo Bolzicco, Maria Silvia Favretto, Ninfa Satariano, Enrico Scremin, Carmelo Tambone, Andrea Tasca

**Affiliations:** 1 Departments of Radiation Oncology, San Bortolo Hospital, Vicenza, Italy; 2 Department of Urology, San Bortolo Hospital, Vicenza, Italy; 3 Department of Medical Physics, San Bortolo Hospital, Vicenza, Italy

## Abstract

**Background:**

Radiotherapy is an increasingly preferred treatment option for localized prostate cancer, and stereotactic body radiation therapy (SBRT) a relatively established modality of therapeutic irradiation. The present study analyzes the toxicity and biochemical efficacy of SBRT in 100 consecutive prostate cancer patients treated with CyberKnife Robotic Radiosurgery System.

**Methods:**

One hundred patients were treated with SBRT at the Radiation Oncology department of San Bortolo Hospital, Vicenza, Italy. All patients included in this IRB-approved protocol-driven prospective study had biopsy-proven prostate cancer. Risk category was low in 41, intermediate in 42, and high in 17 patients. The patients were treated with CyberKnife-SBRT (CK-SBRT), the prescription dose was 35 Gy in five fractions, corresponding to 92 Gy in 2-Gy fractions (α/β =1.5 Gy); 29 patients also received androgen deprivation therapy (ADT).

**Results:**

Median follow-up was 36 months (range, 6–76 months). Acute Grade 2 genitourinary and gastrointestinal toxicity occurred in respectively 12% and 18% of the patients; there were no Grade 3 or higher acute toxicities. Late Grade 1, 2, and 3 genitourinary toxicities occurred in 4%, 3%, and 1% of the patients, respectively; late Grade 1 gastrointestinal toxicity occurred in two patients and Grade 2 toxicity in one patient; no late gastrointestinal toxicities of grade 3 or 4 were observed. Median PSA nadir was 0.45 ng/ml at 36 months for all patients. In the SBRT-monotherapy group, the median PSA nadir at 36 months was 0.62 ng/ml; in the ADT-SBRT group, it was 0.18 ng/ml. Four patients had clinical recurrence: one local, two lymph nodes, and one to the bone. Ninety-six patients had no evidence of biochemical or clinical recurrence. A benign PSA bounce of median 1.08 ng/ml occurred in 12% of the 71 SBRT monotherapy patients at a mean 23 months (range, 18–30 months).

**Conclusions:**

In this study CK-SBRT has provided promising outcomes in localized prostate cancer with good PSA response, minimal toxicity and patient inconvenience.

## Background

The use of stereotactic body radiation therapy (SBRT) in the treatment of localized prostate cancer is supported by recent reports [1-5]. As an alternative to surgery [6], it provides high biochemical control, low risk of complications, minimal duration of treatment, and outpatient treatment opportunity. In the literature, there is evidence that both tumoral and normal tissue response to radiation dose depends on the respective α/β ratios of the said tissues. For prostate cancer, the α/β ratio has been reported to be around 1.5 Gy, lower than the surrounding normal tissues; therefore, the use of a high-dose-per-fraction (hypofractionated) regimen should lead to a more favourable therapeutic ratio [7,8]. Treatment of prostate cancer with high-dose-rate (HDR) brachytherapy is well established: it allows high doses of radiation to be delivered precisely to the target tissue while sparing the surrounding healthy tissue, thus achieving high biochemical control and low toxicity [9,10]. New technologies using image-guided radiotherapy (IGRT) provide a more accurate delivery of the high dose used in SBRT. For instance, CyberKnife® (Accuray Incorporated, Sunnyvale, CA) SBRT (CK-SBRT) delivers hypofractionated treatment regimens with a dose distribution similar to that obtained with HDR-brachytherapy, without the accompanying invasive procedure [11,12].

Here we report our experience with CK-SBRT in which 100 patients with localized prostate cancer were treated. The resulting outcomes and toxicities are disclosed.

## Methods

### Patient eligibility

In June 2006 at San Bortolo Hospital in Vicenza, a prospective protocol-based study for the treatment of localized prostate cancer with CyberKnife Robotic Radiosurgery System was initiated. Since then, 100 patients have been treated (Table 1). The study, approved by the Ethical Committee for Clinical trials of the province of Vicenza, was standard care, with no other intervention. All patients were informed of the benefits and treatment alternatives and gave their written consent.

**Table 1:** Cyberknife®-SBRT: clinical characteristics of 100 patients

**T Stage**	**Patients**
T1c	44 (44%)
T2a-b	29 (29%)
(T2a, 10 pts)	
(T2b, 19 pts)	
T2c	27 (27%)
**Gleason score**	
<6 (2+2, 2+3, 3+2)	8 (8%)
6 (3+3)	76 (76%)
>7 (3+4 11 pts, 4+3 4 pts, 5+5 1 pt)	16 (16%)
**PSA**	
**at diagnosis**	**ng/ml**
All patients	7.72 ng/ml
SBRT (71 pts)	6.48 ng/ml
SBRT+ADT (29 pts)	10.77 ng/ml
**Pre-treatment**	**ng/ml**
All patients	5.03 ng/ml
SBRT (71 pts)	6.31 ng/ml
SBRT+ADT (29 pts)	1.90 ng/ml
**Risk category**	**Patients**
Low (PSA <10, GS 6, T1c, T2a)	41 (41%)
Intermediate (PSA >10, GS 7 or T2b-c)	42 (42%)
High (PSA >20, GS 8–10, 2 Int. risk features)	17 (17%)
**Prostate volume** ( medium 33 cc.)	
≤ 33 cc.	51 (51%)
> 33 cc.	49 (49%)
**TURP** before SBRT (1–16 years)	7 (7%)
**ADT**	
Before SBRT (median 6 months)	8 (27%)
Concomitant and after SBRT (median 12 months)	21 (73%)

SBRT = Stereotactic Body Radiation Therapy.

ADT = Androgen Deprivation Therapy.

TURP = Transurethral resection of the prostate.

Eligibility of the patients was determined by a multidisciplinary tumor board consisting of a urologist, a medical oncologist, and a radiation oncologist. The inclusion criteria consisted of biopsy-proven organ-confined prostate carcinoma without any sign of severe obstruction, an ECOG Grade 0–1, and the informed consent of the patient. All patients were staged with ultrasound-guided biopsy; median prostatic sampling number was of 10 (range, 4–28 samples). The patients with intermediate- and high-risk disease also underwent a bone scan and MRI; the most recent cases received an additional choline PET-CT. Median age was 72 (range, 52–82), with ECOG-performance status value 0–1. As for their risk-category, 41 patients were low risk (PSA ≤ 10 ng/ml; Gleason Score ≤ 6, and tumor category T1c–T2a), 42 were intermediate risk (PSA >10–20 ng/ml or Gleason Score=7 or T2c), and 17 were high risk (PSA >20 ng/ml or Gleason Score >7 or two median risk factors). The clinical stage [13] was T1c in 44, T2a in 10, T2b in 19, and T2c in 27 patients. Gleason Score was <6 (2+2, 2+3, or 3+2) in eight patients and 6 (3+3) in 76 patients; in 16 patients, the score was >6, 3+4 in 11, 4+3 in four, and 5+5 in one patient. Based on the opinion of the urologist, 29 patients received androgen deprivation therapy (ADT), eight before radiotherapy, for a median duration of 6 months (range, 3–8 months), and 21 both before and after SBRT, for a median duration of 12 months (range, 3–36 months). In the final evaluation of freedom from biochemical failure (FFBF) we assessed separately the patients who received ADT or not.

The median baseline PSA was 7.58 ng/ml in the entire cohort, 10.77 ng/ml in the ADT-SBRT group, and 6.28 ng/ml in SBRT-monotherapy group. The median pre-SBRT PSA (pPSA) was 5.03 ng/ml – 1.90 ng/ml in the ADT-SBRT group and 6.31 ng/ml in SBRT-monotherapy group. The mean prostate volume, calculated through transrectal ultrasound (TRUS) immediately before radiotherapy, was 33 cc (range, 15–65 cc). Seven patients had had transurethral resection (TURP), on average 4 years prior to CK-SBRT.

Biochemical failure (BF), defined by a rising prostate-specific antigen (PSA) profile, is an early surrogate of treatment failure. We defined BF in according to Phoenix definition (PSA nadir + 2 ng/mL) (16).

### SBRT technique

**Figure 1:**
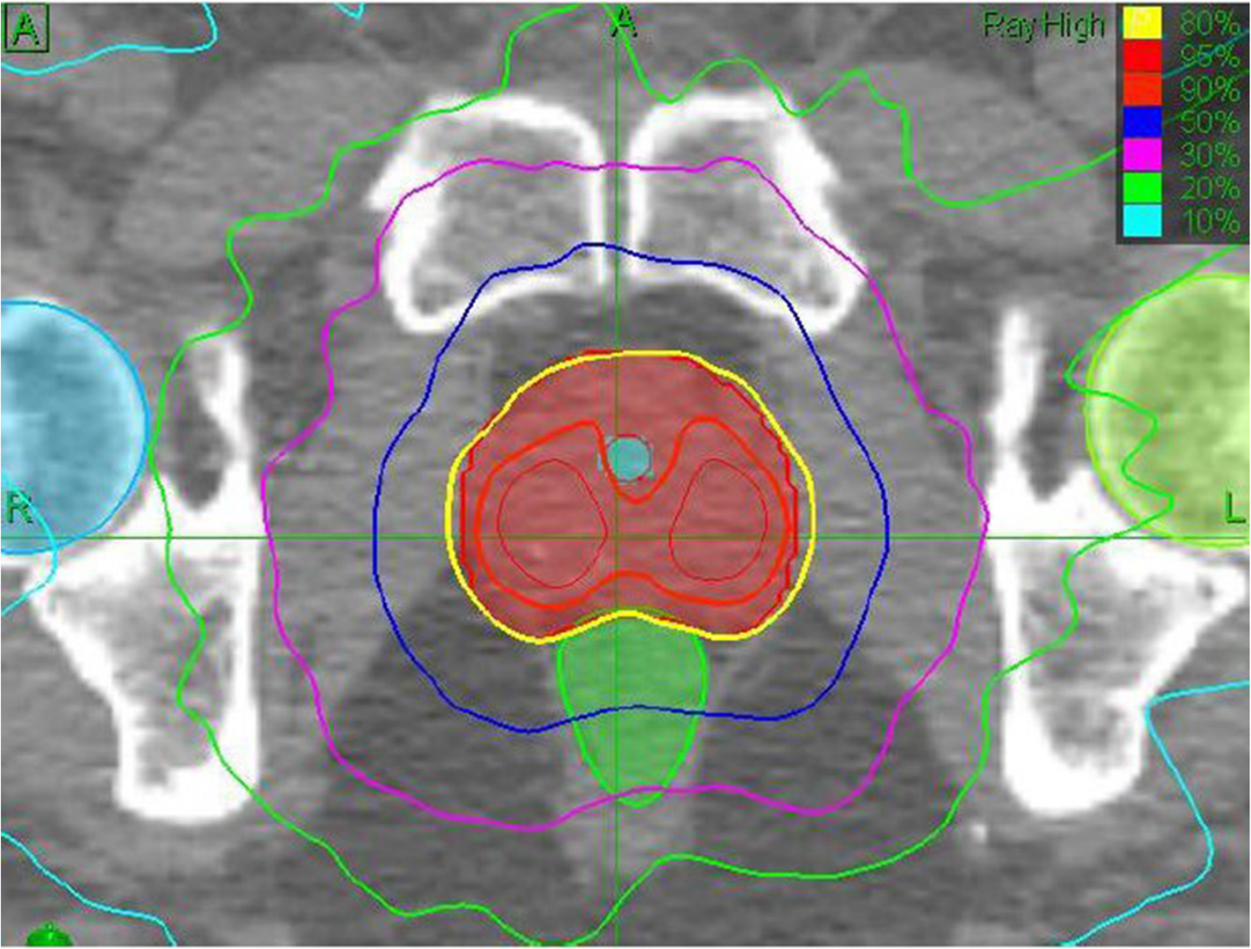
Conventional treatment plane with IrisTM collimator: 35 Gy delivered to the 80% isodose line, tumor coverage 99%, and conformality index 1.22.

Image-guided SBRT was delivered to all patients using the CyberKnife 6-MV linac [3,14-16]. Four gold fiducial markers were implanted transperineally into the prostate under TRUS guidance. After allowing time for the fiducials to settle, usually 10–15 days, treatment planning was performed, using two simultaneous supine axial CT scans with 1-mm slice thickness. An indwelling Foley catheter was used for urethra delineation and precise control of bladder volume; the second scan was conducted with bladder contrast. Patient preparation consisted of a gas-minimizing diet the week before treatment and an empty rectum [17]. After a 3T-MRI scanner has become available in our institution, a pelvic T2-weighted MRI sequence with 0.9-mm slice thickness and gadolinium contrast was performed for each patient, and fused with the planning CT. A total dose of 35 Gy was delivered in five fractions of 7 Gy over consecutive days. The prescription dose covered at least 95% of the planning target volume, normalized to 80% isodose line. The treatments were delivered using a G4 600-MU/min CyberKnife System until the end of 2010, at which time, a G4 800-MU/min was installed, which also included an IRISTM Variable Aperture Collimator and a RoboCouch® (Accuray) (Figure 1).

**Figure 2:**
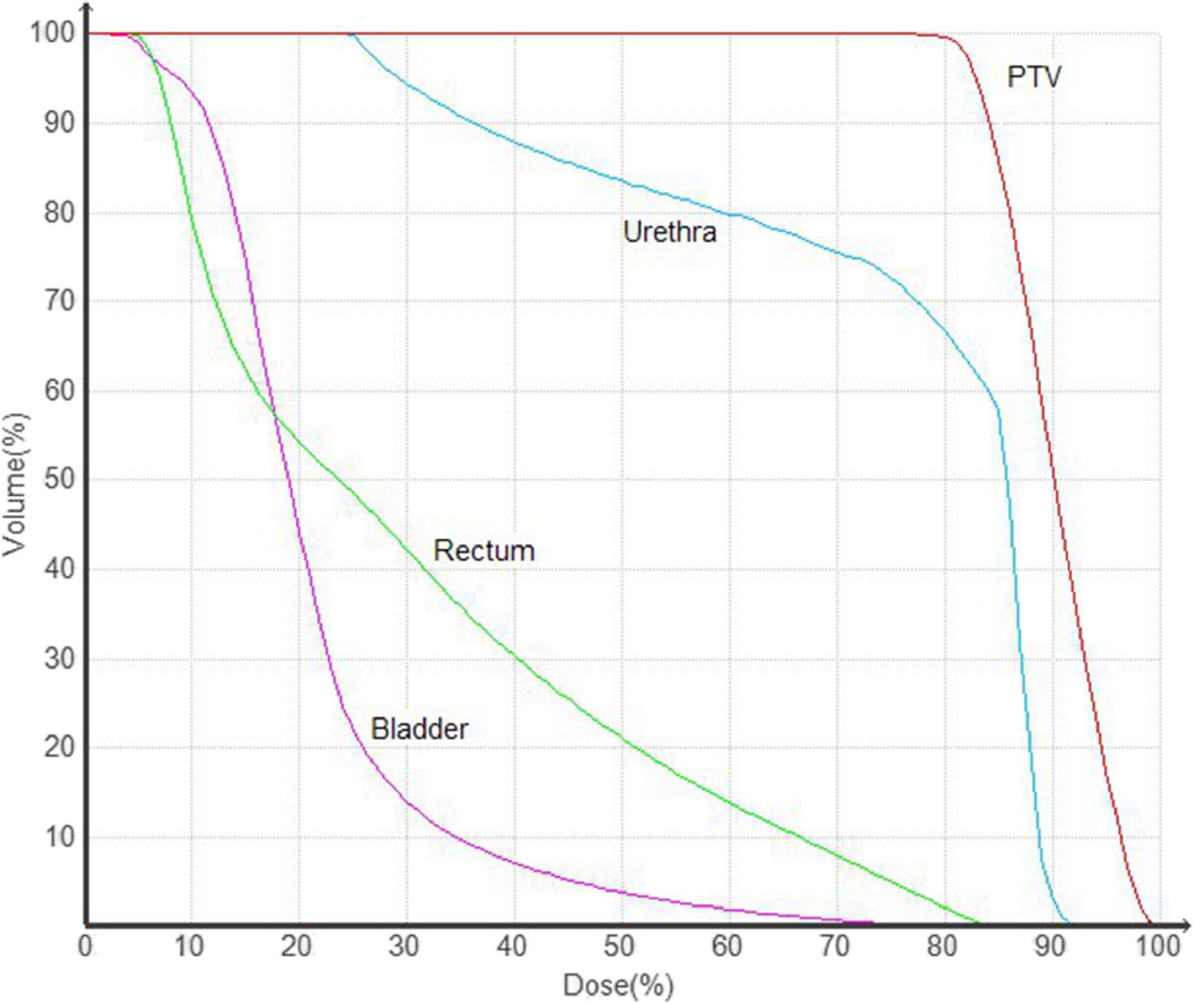
Typical dose volume histogram (DVH) for the target (PTV) and normal tissues (urethra, rectum and bladder).

To optimize treatment planning, the same team of radiation oncologists performed the contouring of the prostate, seminal vesicles, rectum, bladder, penile bulb, bowel, and femoral heads. The prostate and one third of the seminal vesicle volumes were expanded by 5 mm, except posteriorly, where a 3-mm margin was applied. As for the dose volumes, the whole of the bladder, bulb, and femoral heads received 40 Gy to 5%, 29 Gy to 25%, and 25 Gy to 25% of their volumes, respectively; a part of the urethra – a mean 2-cc volume – received 40 Gy to 5% volume; a part of the rectum – mean 50 cc – received 38 Gy to 5%. In Figure 2, several CyberKnife isodose lines are shown in a typical dose-volume histogram.

## Results

### Toxicity

All patients completed the treatment. Median follow-up was 36 months (range, 6–76 months). Acute toxicity was assessed at baseline, and then at 10 days, 1 month, 3 months, and 6 months after CK-SBRT. Acute urinary and rectal toxicities, graded on the Radiation Therapy Oncology Group (RTOG) scale [18], are shown in Table 2.

**Table 2:** Cyberknife®-SBRT: toxicity in 100 patients

**RTOG grade**				
**Acute** (62 pts)	I	II	III	IV
Urinary	34%	12%	-	-
Rectal	27%	18%		
**Late** (9 pts)				
Urinary	4%	3%	1%	
Rectal	2%	1%	-	-

SBRT = Stereotactic Body Radiation Therapy.

RTOG Grade= toxicity based on Radiation Therapy Oncology Group.

The most frequent acute complaints were urinary frequency/urgency, stool frequency/pain and rectal urgency, usually during the first week, or at most 2 weeks after treatment. Sixty-two patients had acute toxicity: 34% had Grade 1 and 12% Grade 2 urinary toxicity; 27% had Grade 1 and 18% Grade2 gastrointestinal toxicity. No Grade 3 or 4 acute urinary or gastrointestinal toxicities occurred. Acute toxicity was usually resolved within 1 month of radiotherapy on basic symptomatic therapy. When considering pre-treatment prostate volume, there was no meaningful difference between the 51 patients with prostate volume ≤ 33 cc and the 49 patients with prostate volume >33 cc, with 18% and 28% acute toxicity rates, respectively.

Late urinary toxicity (at 6 months or later post-CK-SBRT) occurred in 8% of the patients: 4% grade 1, 3% grade 2 and 1% grade 3; the main problems were urgency/frequency (Table 2). The patient with Grade 3 toxicity had undergone urologic tests, including cystoscopy and urethral dilatation. Three of the seven patients who had had TURP before treatment experienced late urinary toxicity with the percentage distribution 1% Grade 1, 1% Grade 2 and 1% Grade 3. Two per cent of the patients experienced Grade 1 late rectal toxicity, i.e. occasional blood in stool, and one patient had Grade 2 in the form of frequent-daily bowel movements. We have not seen any other toxicities.

### PSA response

**Figure 3:**
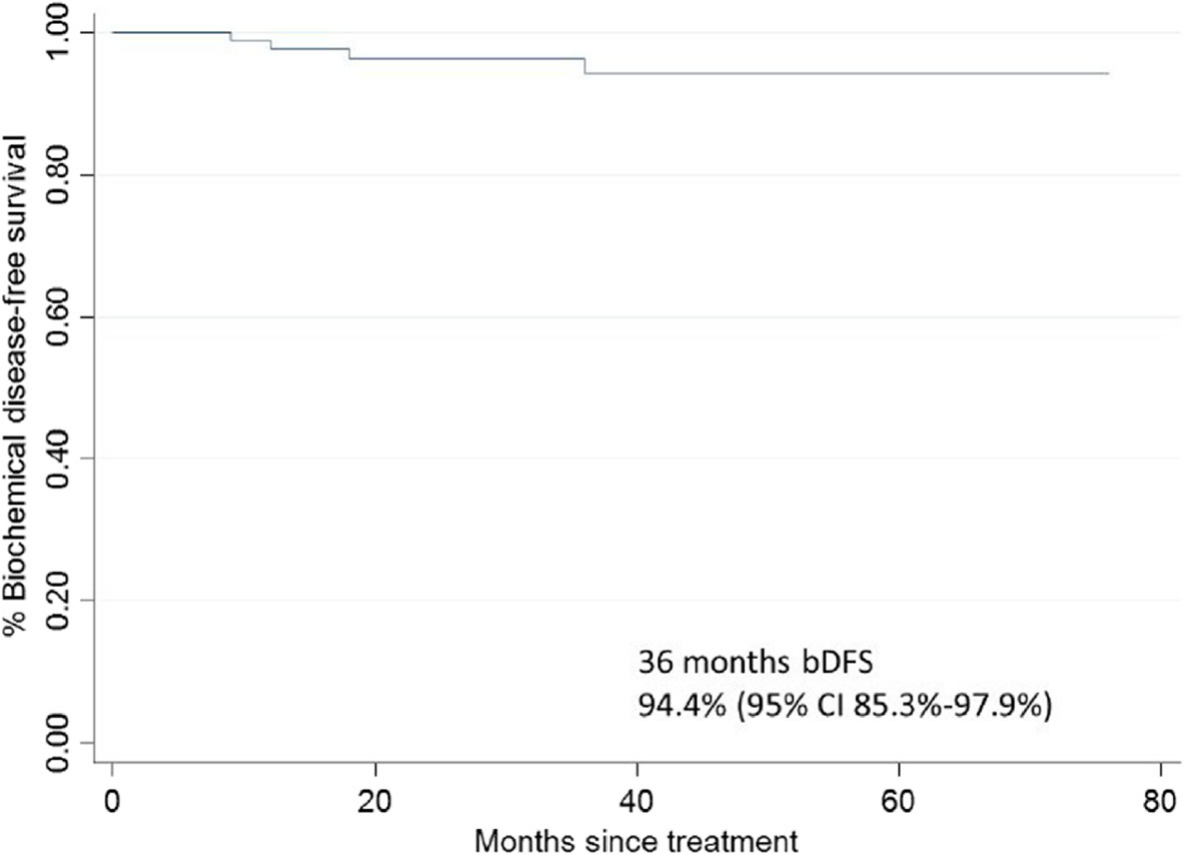
Kaplan-Meier biochemical disease-free survival curve in 100 SBRT-patients for prostate cancer.

**Figure 4:**
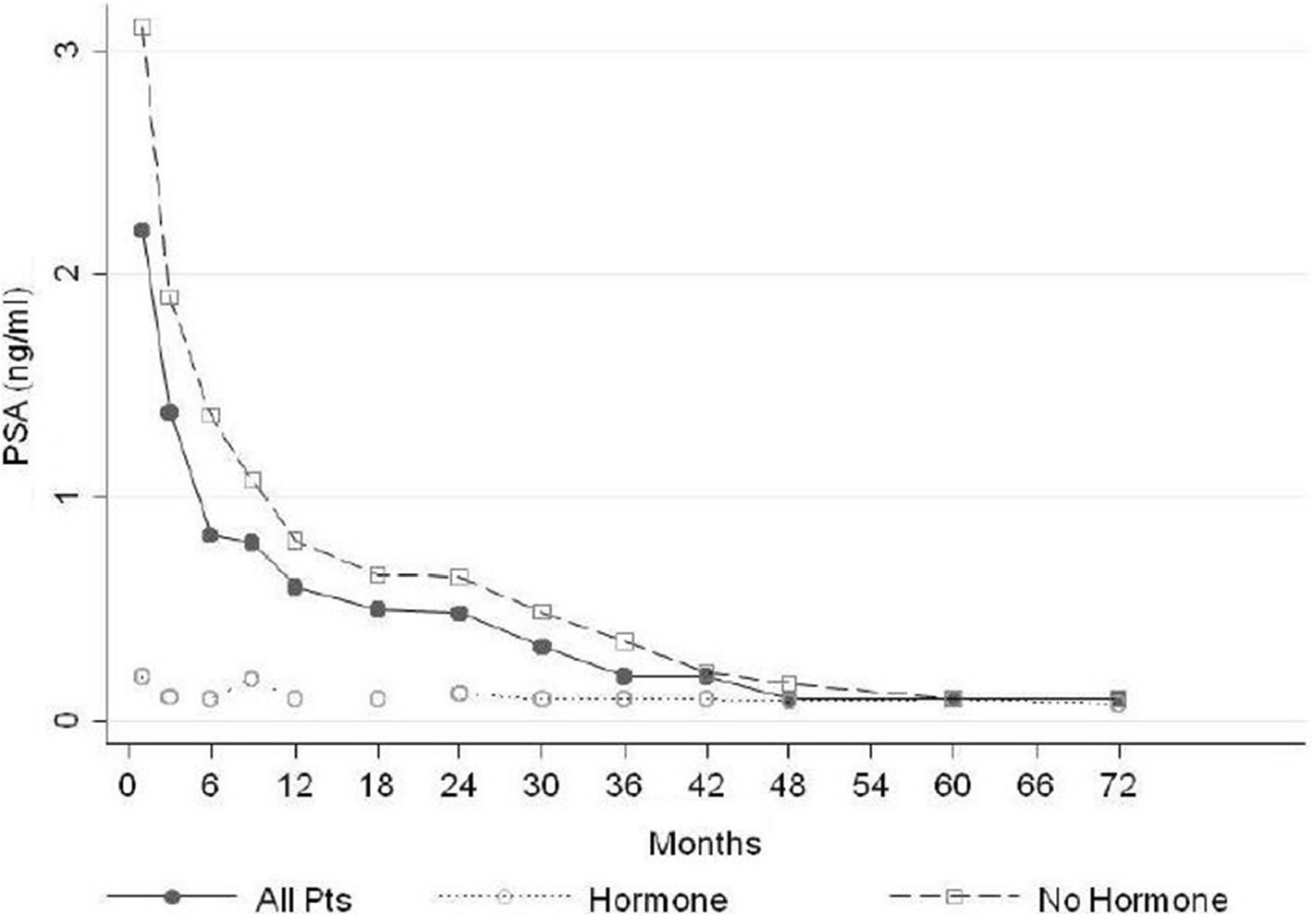
Mean PSA level (ng/ml) for all patients, patients who did not recive hormone therapy and patients who recived hormone therapy.

The 3-year biochemical progression-free survival rate was 94.4% (95% CI= 85.3%–97.9%) (Figure 3). A gradual decline in PSA levels through first, second, and third years occurred, with the median nadirs of 0.73 ng/ml, 0.67 ng/ml, and 0.45 ng/ml, respectively. Considering the 71 CK-SBRT monotherapy patients and the 29 patients who also received ADT separately, both had steadily decreasing PSA nadirs. The SBRT monotherapy patients had the PSA nadirs of 0.93 ng/ml, 0.87 ng/ml, and 0.62 ng/ml at 1, 2, and 3 years; and the adjuvant ADT patients had 0.26 ng/ml, 0.30 ng/ml, and 0.18 ng/ml at the same time points, respectively (Figure 4).

Sixteen of the 29 ADT patients were able to discontinue the therapy at least 1 year before the publication of this article (range, 12–59 months); no biochemical failures were observed among these patients with a median follow-up of 40.3 months. At 2 years, there was no difference between low- and intermediate/high-risk patients, with PSA readings of 0.44 and 0.50 ng/ml, respectively (p = 0.63). A benign PSA bounce – a PSA uptick and a subsequent decline to nadir – was observed in 12% of the 71 SBRT-monotherapy patients (Table 3). The median value of the PSA bounce was 1.08 ng/ml and it occurred at a mean 23 months (range, 18–30 months).

**Table 3:** CyberKnife® 71 SBRT-monotherapy patients

	**Patients**	**ng/ml**	**months**
PSA Bounce	12%	medium value 1.08	medium 23 (18–30)

SBRT = Stereotactic Body Radiation Therapy, Bounce= a PSA rise and a subsequent decline of nadir.

According to the Phoenix definition [19], four patients – three intermediate- and one high-risk – had biochemical relapses at respectively 9, 12, 18, and 30 months post CK-SBRT: one patient had local, two patients nodal, and one patient distant (bone) recurrent disease. All relapsed patients received hormone and/or chemotherapeutical treatments and were alive at a median follow-up of 14 months, range 6–18 months, from salvage therapy.

## Discussion

The advantages of hypofractionation (decreased fraction number with increased dose per fraction) in prostate cancer treatment are supported by recent literature [20-22], based on the presumably low α/β value of prostate cancer cells [2,7]. There is evidence of the superior radiobiological effects of radiotherapy in higher-dose fractions [23,24]. The use of SBRT is supported by convincing studies [2,4,5] on the clinical results of hypofractionation. Fuller et al. [11] have compared HDR brachytherapy plans with CK-SBRT plans in 10 patients for the same dose and fractionation scheme, favouring the use of CK-SBRT as it spared healthy tissues, such as the urethra, while simulating the delivery of an HDR brachytherapy-like dose distribution to the target. Fuller’s was one of the initial studies, after which several clinical reports have supported the use of CK-SBRT in low- to intermediate-risk prostate cancer treatment.

The present study is an update of our preliminary CK-SBRT experience [17] of 45 low- to intermediate-risk prostate cancer patients. Since then, 16 high-risk patients were also treated in our department. In the present study, they were separately analyzed to assess whether SBRT was an appropriate treatment for prostate cancer patients who cannot have surgery. Katz et al. [25] have reported on the treatment of 12 patients with high-risk prostate cancer with a daily dose of 7.25 Gy delivered in five daily fractions, with a 4-year actuarial (FFBF) of 75%. Kang et al. [26] have treated 29 high-risk patients with daily fractions totalling 32–34 Gy in a four-fraction regimen, resulting in an FFBF of 90.8% at last follow-up. Among our 16 high-risk patients, with a medium follow-up of 36 months, one biochemical recurrence occurred. Six of these patients also received ADT, two were still on therapy at last follow-up; the patient with biochemical recurrence did not receive ADT.

The median planning target volume (PTV) was 67 cc. (range, 46–109 cc), including a 5-mm margin in all directions except posterior, where the margin was 3 mm. The median prostate volume was 33 cc (range, 15–69 cc). Usually, patients with a prostate volume > 60 cc are excluded from SBRT studies, following the example of HDR brachytherapy [27]. Two patients with prostate volumes > 60 cc were treated at our institution, without any higher toxicity.

The prescription dose for all patients was 35 Gy to the 80% isodose line in five consecutive fractions, corresponding to a normalized total dose (NTD) of 92 Gy if delivered in 1.80-Gy fractions, assuming an α/β ratio of 1.5 Gy. In the literature, the prescription dose varies from 32 Gy in four fractions to 38 Gy in four fractions [25]. Katz et al. [28] have used doses of 35 Gy in five fractions and 36.6 Gy in five fractions, reporting no FFBF difference between the two groups and a non-statistically-significant higher urinary late toxicity in the higher-dose group. Freeman and King [29] have compared 35 and 36.25 Gy doses in five fractions in 41 patients with similar conclusions.

Although the dose to the testicles was not constrained in our treatment planning, a random check of 20 patients showed a median testicular dose of 4 Gy; Katz et al. [2] have reported a median D50 testicular dose of 5.28 Gy (range 3.2–11.8 Gy) in 12 CK-SBRT patients. Oermann et al. [30] have published a study on 26 patients treated with CK-SBRT, where serum testosterone levels and endocrine changes were observed: a small decline in total testosterone levels without the endocrine changes, typical of hypogonadism, was reported.

In the present study, treatment planning consisted of four gold fiducial markers placed in the prostate to verify organ position in real time and render prostate’s spatial geometry. Use of a urethral catheter was standard practice in our CK-SBRT patient preparation, although some other institutions may not use this procedure: in fact, one might argue that a distended bladder would avoid higher isodose-lines. In our protocol the bladder receives, based on capacity, an average of 100 cc saline, thus preventing over-distension, which may have a negative effect on target position. The aim of this technique is to try to keep the bladder volume constant during therapy, as also stated in Viswanathan et al. [31]. We also use the urethral catheter to discern the outline of the prostatic urethra perfectly. MRI was used in the treatment planning of 30 of the 100 patients, without any other procedural or planning changes. 3T-MRI was used in CT-MRI treatment planning and staging to avoid certain common staging errors [32] that have been made in surgical series of T1 and T2 patients, which have statistically led to over staging about 40% of patients [33].

In the present study, Grade 2 acute toxicity was found in 30% of the patients – 12% genitourinary and 18% gastrointestinal – thus reproducing the results of our previous study [17] in which, in a total number of 45 patients, we had reported 35% acute toxicity, with no grade 3 or 4 acute urinary or gastrointestinal toxicities. On analysis of the patients treated with and without urethral catheter, acute urinary toxicity was 46% and 50% respectively, with only 10 patients having been treated without catheter. As for gastrointestinal toxicity, Grade 2 acute rectal toxicity occurred in 18% of the patients, all toxicities resolving with minimal medication. No Grade 3 or 4 acute rectal toxicity occurred. Our results were comparable with those in the literature: Jabbari et al. [12] have observed acute rectal Grade 2 toxicity in 5% of the 38 SBRT-treated patients, whereas Kang et al. [26] have reported 9.1% Grade 2 acute rectal toxicity in the 44 patients they treated.

As for late toxicity in the present series, our 5% of Grade 2 or 3 late urinary or rectal toxicity rate is comparable to the literature: Katz et al. [28] have encountered 9.7% late genitourinary or rectal toxicity in 41 patients; Freeman et al. [29] had 12% late urinary or rectal toxicity in 41 patients; Mc Bride et al. [3] observed late urinary Grade 2 toxicity in seven patients (17%) and Grade 3 toxicity in one patient (2.2%), while three patients (7%) experiencing late Grade 2 rectal toxicity and two patients (5%) late Grade 3 proctitis.

When comparing other prognostic factors, e.g., ADT, in relation to acute and late genitourinary toxicities, no differences were seen. Genitourinary acute toxicity occurred in 44.8% and 46.4% of the ADT-SBRT group and SBRT-monotherapy group patients, whereas late genitourinary toxicity occurred in 3.4% and 9.8%, respectively, thus confirming the lack of influence of ADT on toxicity in patients treated with SBRT. When evaluating the results of patients treated with the earlier vs. the newer CyberKnife System, i.e. G4, as for late urinary or rectal toxicity, the former had 5.1% (4/78) Grade 2–3 toxicity, while in the latter group there was no case of toxicity; as the late group’s median follow-up is only 12 months, the data need further confirmation. We also analysed the incidence of late genitourinary toxicity in the seven patients previously treated with TURP, which showed three patients with Grade 1–3 toxicities. The patient who had had TURP 4 years before CK-SBRT had late Grade 3 genitourinary toxicity, which completely resolved at 2 years post treatment.

The results of the present report appear to be favourable on CK-SBRT in the treatment of localized prostate cancer, with the encouraging actuarial freedom-from-relapse rate of 96% at 3 years. We had four relapses in 100 patients, three cases with intermediate-risk and one with high-risk disease. Katz et al. [16] had a 1.9% relapse rate in 254 patients: two failures in low-risk and three in high-risk patients. Friedland et al. [4] have reported 2.6% (3/112 patients) of failures. The PSA results in our group of 100 patients decreased with a nadir of 0.54 ng/ml at 36 months, which may be considered a satisfactory outcome. Among the recurrence-free low-risk-patients treated with SBRT-monotherapy (41 patients), the median PSA nadir was of 0.47 ng/ml (range, 0.02–2.05) at 36 months. King et al. [1] have reported a PSA nadir of 0.32 ng/ml (range, 0.03–2.65 ng/ml) at 33 months in a series of 41 low-risk-patients; in Katz et al. [28], reported a PSA nadir was 0.2 ng/ml in 71% of 82 patients with a four years median follow-up. Considering both the ADT-SBRT (29 patients) and SBRT-monotherapy groups (71 patients), we observed a nadir of 0.16 and 0.75 ng/ml at 36 months, respectively. The nadir PSA at 36 months in non-recurrent patients treated with ADT-SBRT according to their risk categories were, respectively, 0.14 ng/ml and 0.17 ng/ml in low- (7 patients) and intermediate-high-risk patients (22 patients). In the SBRT-monotherapy group, the average PSA was, respectively, 0.54 ng/ml and 0.76 ng/ml in low- (34 patients) and intermediate-high-risk patients (33 patients). Nine of the 71 SBRT-monotherapy group of patients (12.6%) experienced a median benign PSA bounce of 1.08 ng/ml. The median time to PSA bounce was 23 months (range, 18–30 months). King et al. [1] have observed a PSA bounce in 12 of their 41 patients (29%), Katz et al. [2] a PSA bounce in 16% of the patients (37/237) at a median of 18 months, with a median value of 0.35 ng/ml (range, 0.2–1.08 ng/ml); McBride et al. [3] have reported a median PSA bounce of 1.07 ng/ml at a median time of 11.6 months in nine of the 45 patients (20%). In our opinion, the bounce, normally occurring during the first 2 years from treatment, is not prognostic of a recurrence.

## Conclusions

Although the advent of SBRT is quite recent, and therefore needs further validation with randomized clinical trials, so far it seems to be a safe, easy, and effective therapy for localized prostate cancer, improving patient convenience. The advanced technology incorporated into the CyberKnife System enhances the possibility of achieving a more precise targeting with lower toxicity. Our 5-year progression-free survival rate of 93% seems to compare favourably with the results obtained with surgery, LDR, or HDR brachytherapy.

## Competing interests

The authors declare that they have no competing of interest.

## Authors' contributions

GB and MSF conceived of the study, participated in its design and coordination and drafted the manuscript. NS performed the treatment plan. ES and CT implanted the fiducials and attented to the patients. AT participated in the design. All authors read and approved the final manuscript.

## Pre-publication history

The pre-publication history for this paper can be accessed here:

http://www.biomedcentral.com/1471-2490/13/49/prepub
